# Fire ecology database for documenting plant responses to fire events in Australia

**DOI:** 10.1038/s41597-025-04705-6

**Published:** 2025-03-07

**Authors:** José Rafael Ferrer-Paris, Ada Sánchez-Mercado, William K. Cornwell, Mark Ooi, Mark Tozer, Berin D. E. Mackenzie, Renee Woodward, Andrew J. Denham, Tony D. Auld, David A. Keith

**Affiliations:** 1 School of Biological, Earth and Environmental Sciences, University of New South Wales, Sydney, Australia; 2 UNSW Data Science Hub, University of New South Wales, Sydney, Australia; 3 IUCN Commission on Ecosystem Management, Gland, Switzerland; 4 Department of Climate Change, Energy, the Environment and Water, Sydney, New South Wales Australia; 5 University of Wollongong, Wollongong, Australia

**Keywords:** Fire ecology, Forest ecology

## Abstract

An understanding of fire-response traits is essential for predicting how fire regimes structure plant communities and for informing fire management strategies for biodiversity conservation. Quantification of these traits is complex, encompassing several levels of data abstraction scaling up from field observations of individuals, to general categories of species responses. We developed the Fire Ecology Database to accommodate this complexity. Its conceptual framework is underpinned by a flexible data pipeline enabling links between fire-related trait data and event information at individual, population, and community levels. Key features include: (a) concise and documented trait and method vocabularies; (b) documented uncertainty in observations and aggregation; and (c) documented origin of data including field observations, laboratory experiments, and expert elicitation. We demonstrated application of our framework using data from new field surveys and existing data sets in New South Wales, Australia. The database includes 14 traits for 6,287 plant species derived from 8,936 field work records from 2007 to 2018, 7,054 field records from surveys after 2019, and 48,306 records from 301 existing sources.

## Background & Summary

Fire regimes are recurring disturbances that vary in frequency, intensity, season, and type^1^, with each fire event affecting patches of different size and forming landscape mosaics of variations in fire histories. Fires mediate coexistence and diversity of species over large portions of the earth’s land surface^2–4^. Many plants have evolved morphological, ecophysiological, and life history traits that enable their persistence under particular fire regimes^1,5–7^. Examples include heat-protective tissues, regenerative organs, post-fire flowering, seed dormancy mechanisms and fire-stimulated germination^8–10^.

An understanding of fire-response traits has proved useful for predicting how fire regimes structure plant populations and communities^6,11,12^, and for informing fire management strategies for biodiversity conservation^13,14^, particularly where changed fire regimes can lead to biodiversity decline^15^.

Research and management applications have generated a substantial demand for fire-response trait data, with several compilations emerging semi-independently from researchers and land management agencies working in different ecosystems or geographic regions^12,16–20^. Interpretation of plant traits related to fire response is complex, in part because their functional outcomes depend on multiple ecological processes that interact across scales^6^. Also, relevant evidence comes in different formats that may have undergone several steps of data abstraction from field observations through interpretation and re-interpretation in subsequent publications. In addition, trait values vary in relation to heritable characteristics (variation within and between populations), growth stages, fire properties (event- and interval-related), and spatio-temporal environmental variability e.g.^8,21^. This complexity underscores the need to interpret traits in their ecological context, with an associated need to record spatially and temporally explicit trait observations with relevant covariables.

The definition of fire-response traits is a foundational step that determines the scope of the data model and any potential constraints on applications. Most existing data models^12,16^ record traits in dichotomous or categorical format as a species-level property. Attributing single trait values for each species precludes quantitative trait data where it is available. This not only limits the description (and hence understanding) of trait variation related to genetic differences, growth stages, fire properties and environmental characteristics, but it also constrains the ability to transparently document, report on, and analyse uncertainties in the observational or elicited data^22^. There have been attempts to resolve intraspecific variation in fire-response traits^17^, and recent approaches have developed flexible data models that accommodate plant trait data at different levels of specificity^23^.

Interpretation of traits can add additional levels of abstraction, with some authors combining one or more traits into a category of expected species responses. For example, the Vital Attribute model^11^ assigns species to groups based on combinations of species traits from multiple life stages (reproductive adults, juveniles, and propagules) to describe contrasting strategies of plant persistence or recolonisation after fire disturbances. Thus, variation in the expression or measurement of individual traits could cascade to the derived species attributes in different ways.

Here, we introduce the Fire Ecology data pipeline and database (FEDB). We describe how they address limitations associated with data abstraction, definition of traits, their multiple expressions (as binary, categorical, or continuous variables), relationships among variables, and derived traits. Resolving these issues in a systematic data model helps to frame an approach for improved data collection. While other databases focus on fire-related plant traits, our database expands on this by incorporating how species as a whole respond to fire events, including changes in population size and structure, and community properties following fire events, providing a more comprehensive resource for understanding the ecological impacts of fire on plant communities.

Our approach includes core features that allow flexibility and robustness to support dynamic data assets that may be adapted as trait concepts and methods develop. Key innovations of the data model include: (1) trait definitions and relationships that link units of observation at different levels of organisation, including individuals, populations, species and ecosystems; and (2) the ability to store and report on spatially and temporally explicit trait observations linked to fire events. Other features include: (3) comprehensive and accessible trait and method vocabularies to enable clear and reproducible interpretation of numerical and categorical data; (4) data structures and vocabularies that document uncertainties in observations and aggregation; (5) documented origins of data records enabling steps of data abstraction to be traced; (6) links to one or multiple taxonomic repositories e.g.^24,25^, ensuring up to date species names; and (7) data protocols and proformas that allow users to align data collection strategies and effort with available resources.

The data model ensures that uncertainties in estimates of trait values are documented, appropriately propagated, summarised, and reported to users for decision making through three enabling features: (a) methods of observation are recorded for every trait (e.g. direct observation, inference based on related taxa, expert elicitation, etc.); (b) bounded estimates can be stored for quantitative traits (upper, lower, best estimates) or multiple plausible categories for categorical traits; and (c) an ability for the administrator to weight information sources according to their apparent reliability for queries and reports.

Here, we document the data pipeline and the structure of the database, focusing on an initial set of 14 priority traits. We applied this framework to a case study (New South Wales, Australia) with diverse data contributions including: (a) direct field observations from surveys undertaken within 24 months after known fire events in systematic sample plots; (b) time series data from an extended field experiment; and (c) two existing databases that compile plant trait data from multiple primary sources^18,23^. Additionally, we linked the records to a regional taxonomic species list^26^.

This case study demonstrates the integration of a large volume of legacy data from existing sources along with new observational data. Our database not only serves as a repository for fire-related traits but also facilitates exploration of the broader ecological consequences of fire regimes. By linking trait data with species responses to fire, it enables fire management strategies to be more precisely tailored to the ecological needs of different plant species and communities. The general framework and data model is applicable globally and should serve a diversity of research and management needs.

## Methods

### Fire-related plant traits

By considering plant life stages (seed, seedling or standing plant) and life cycle processes (survival, growth, reproduction, germination, recruitment, and dispersal), we defined an initial set of 38 traits to describe responses of plant species to fire (see Online-only Table 3). Of these 38 traits, we selected 14 priority traits (Table 1) based on their relevance to fire management (as prioritised by conservation agency staff) and the availability of data^1,6,8,11,16^. The first release of the Fire Ecology Database (FEDB, version 1.1) includes the priority traits, while the remaining 24 will be added in future releases.

**Table 1:** Fire-related traits to describe responses of plant species to fire.

Trait name	Trait code	Description
*Dispersal*/*Seed*		
Propagule dispersal mode	**disp1**	Categorical. Propagule dispersal mode.
*Germination*/*Seed*		
Seedbank type	**germ1**	Categorical. Location & persistence of seedbank.
Seed dormancy type	**germ8**	Categorical. Seed dormancy type
*Growth*/*Standing plant*		
Age to develop regenerative/resistance organs	**grow1**	Numerical. Developmental age for fire- regenerative or resistant organs (Age at which half an even-aged cohort can survive full scorch).
*Recruitment*/*Seedling*		
Establishment pattern	**rect2**	Categorical. Seedling establishment pattern in relation to mature vegetation.
*Reproduction*/*Standing plant*		
Post-fire flowering response	**repr2**	Categorical. Strength of flowering response after fire events.
Age at first flower production (from seed)	**repr3**	Numerical. Age at first flower production (years).
Time to first postfire reproduction (from resprouts)	**repr3a**	Numerical. Time to first reproduction after fire for resprouting taxa (years).
Maturation age	**repr4**	Numerical. Years taken for 50% of an even-aged cohort to produce their first viable seed.
*Survival*/*Standing plant*		
Resprouting - full canopy scorch	**surv1**	Categorical. Proportion of individuals surviving & resprouting after full canopy scorch.
Regenerative organ	**surv4**	Categorical. Regenerative organ type.
Standing plant longevity (max)	**surv5**	Numerical. Maximum life span *in situ*.
*Survival*/*Seed*		
Seedbank half-life	**surv6**	Numerical. Time taken for half of an even-aged seed crop to die/decay *in situ*.
Seed longevity	**surv7**	Numerical. Maximum life span *in situ*.

These 14 traits populated with data in the initial release for NSW are a subset prioritised from a larger set of 38 traits to be implemented in future releases of the database (see Online-only Table 3 for full trait list).

We developed descriptions and defined the range of valid values for each trait by reviewing current literature and our field experience^5,8,27–29^. Traits took either categorical values (e.g., type of regenerative organ), or quantitative values of response (e.g., time in years).

### Outline of data pipeline

Ideally, values of the fire-related plant traits are attributed to populations or species based on field observations, measurements and calculations. However, most of the current knowledge is inherited from existing data sources, and the original data processes and interpretations might be partially undocumented or untraceble.

**Fig. 1:**
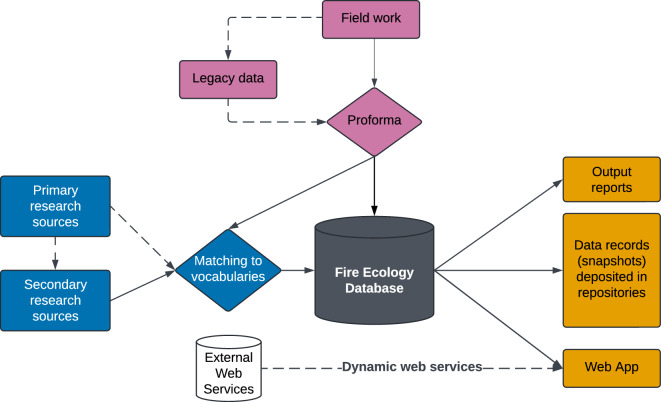
Data pipeline in the Fire Ecology Database (FEDB). The two main data streams, direct field observations (purple) and existing data sources stream (blue), are integrated in the FEDB. Primary research sources include records summarising field observations, and secondary research sources includes records extracted from other sources. Users can access this information through reports, data records, or the Web App.

We designed a robust data pipeline with two main data streams to provide a broad coverage of these traits and accommodate both structured field records (raw data) and estimated trait values (derived data) from existing sources (Fig. 1). The documentation of all steps of data ingestion, transformation and storage helps to avoid data degradation and enhance the reusability and interpretability of the trait data^30^.

The first data stream focuses on the standardisation of direct field observations of plant responses at survey locations (sites). Data aggregated from this stream can later be used to calculate or summarise traits per population or species in a spatial and temporal context. The second data stream imports and harmonises estimated trait values (already processed or interpreted by the original authors) from scientific literature, reports or unpublished sources. These records provide a baseline of trait values for species under-represented in the field surveys.

These two data streams have different requirements for data organisation but are connected through the use of shared vocabularies and taxonomic arrangement. The FEDB serves as a gateway to combine these data streams and provide outputs for different use cases. The general structure of the database was developed prior to data collection, and refined progressively as needed to accommodate practical requirements and user requirements.

The following sections are based on the selected case study (plants of New South Wales, Australia), but the general concepts and approach are applicable to other regions of the world. We share all code and records transparently and invite users to reuse and adapt the data and software for other case studies.

### Database structure

**Fig. 2:**
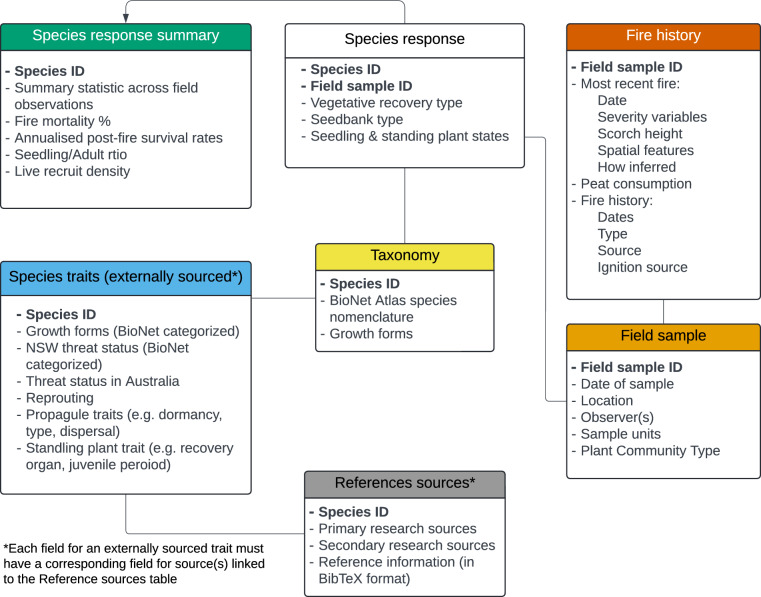
Modules of information in the Fire Ecology Database: species taxonomy, references to data sources, species-level traits from published and unpublished sources, original field observations on population and species traits, fire event characteristics, sampling event, and derived traits from field observations/samples. Primary or foreign key fields are in bold font.

The Fire Ecology Database was designed to combine seven modules of information relevant to the two data streams: (1) species taxonomy; (2) references to data sources; (3) species-level traits from published and unpublished sources; (4) original field observations on population and species traits; (5) fire event characteristics; (6) sampling event; and (7) derived traits from field observations/samples (Fig. 2). This structure was implemented as a relational database with several tables organised in three main schemata: one for the taxonomic arrangement (equivalent to module 1 above); a second for the data derived from the existing sources (modules 2 and 3); and one for the field data (modules 4 to 6; see Online-only Figure 10). Species level summaries of traits derived from the field data (module 7) can be implemented internally as temporary tables or views, or externally using scripts to query, summarise, and transform the data.

The taxonomy data schema includes one table with a master taxonomy that handles all relevant taxonomic nomenclature and additional species information. For this case study, the species nomenclature follows the BioNet Atlas for New South Wales^31^. The species list queried in August 2024 includes 16,157 names for vascular and non vascular plant taxa. They are 11,027 current scientific names for species-level taxa and 1,503 for infra-species taxa (subspecies, forms or variants). The rest are synonyms, homonyms and other invalid names. For taxonomic decisions on plants, BioNet uses the following sources in order of precedence: NSW Biodiversity Conservation Act 2016 (https://legislation.nsw.gov.au/view/html/inforce/current/act-2016-063), PlantNet^26^, Australian Plant Name Index^32^, and the International Plant Name Index^33^. All data ingested into FEDB used species codes that are fully compatible with the BioNet Atlas nomenclature, allowing automatic links to update older names and resolve synonyms.

The data schema for existing data sources has as many tables as required to record multiple traits as reported in published or unpublished sources. For the current release (version 1.1) there are 14 tables, but additional tables will be added to expand the number of traits in future releases. Each trait is recorded in a different table to allow the application of data type constraints (via controlled vocabularies) for each trait, but the structure of the tables is similar to facilitate concatenation of data for export and visualisation (Online-only Figure 10). Trait tables differ depending on the type of data recorded, and on the methods of estimation used. All trait tables require a taxonomic name and code, and a ‘raw’ value imported verbatim from the input source when possible. Qualitative or categorical traits use a unique controlled vocabulary to constrain the normalised value field (‘norm value’). Each item of the vocabulary has a short description of its meaning in this context to facilitate manual curation. Quantitative traits use a triplet of values (upper and lower bounds encompassing a best estimate) to describe a fuzzy number^34^ that describes the uncertainty in the measurement or estimation of the trait. Most traits have an additional field describing the methods of inference of the values (e.g. direct observation, inference drawn from different forms of evidence, etc.). Two fields incorporate comments verbatim from the input source and comments added after import of the record, respectively. Other fields assign weights and weighting rationale to a record, for example, to account for variation in data reliability (i.e. downweighting less reliable records). The input source and any additional references linked to the record are recorded in separate fields.

The field observation schema includes seven tables to arrange surveys, field sites (spatial information), field visits (temporal information), fire history, vegetation structure, field samples, and species per sample, as well as ancillary information including unique observer identity codes (Online-only Figure 10). All data in the field schema are linked to a field site or a field visit. The field site table defines a location with geographical coordinates (using the Simple Features for SQL, SFSQL, specification of a geometry column), while the field visit table defines each unique occasion when the field site was sampled. Thus, one field site can be visited one or more times, and multiple related field visits are grouped in a survey described in the survey table. Fire history information obtained from *in situ* observation or from reference to external records is linked to field sites. This can help to plan multiple visits to track a temporal sequence of plant responses to fire. The vegetation structure and field samples are linked to a particular field visit (Online-only Figure 10).

### Data input - field observations

Between October 2019 and December 2021, a total of 94 field sites were visited at least once in eastern New South Wales (Table 2). We also adapted data from 66 field sites in semi-arid mallee woodlands in southwestern New South Wales surveyed between 2007 and 2018 after experimental fire treatments including published^35^ and unpublished data (Table 2).

**Table 2:** Summary of data inputs (see Methods text for details).

Type	Unit of observation	Spatial information	Number of records	Number of taxa (including non-native)	Data source
**Primary Observations**					
Post-fire field surveys	Individual	94 sites	8936	765	East coast post-fire surveys 2020–2022
Time series field observations	Individual	66 sites	7054	129	Mallee vegetation dynamics 2007–2018^35^
**Compilations**					
Fire response	Species	Not applicable	9486	2549	203 sources compiled in NSW plant fire response database^18^
Species traits	Individuals/Populations/Species	Variable	39820	6173	153 sources compiled in AusTraits plant database^23^
Total				6287	

Each visit was conducted by a main observer and one or more additional observers. Observers recorded location and site features, including geographical coordinates, elevation, substrate type, and vegetation class^36^. Sampling units referred to as “subplots”, are nested within each site. Each visited site could have one or more subplots with a size appropriate to the vegetation type (e.g. 1 × 1 m for peatlands - 20 × 20 m for forests).

The date (as a range between earliest and latest plausible values) and cause of ignition (lightning, prescribed burn, accident, arson, cultural burning, unknown) were estimated for the most recent, penultimate, and antecedent fires based on records held by government agencies, *in situ* evidence (e.g., fire scars on vegetation) and personal communications from land holders and fire managers. Time since last fire is calculated for each visit within the database as the time between the last known fire and the date of field visit. For sites surveyed within 12 months of fire passage, three fire severity variables were estimated *in situ* in all subplots combined: i) maximum scorch height; ii) percentage of pre-fire foliage consumed, scorched and unburnt in each of four vertical vegetation strata where present (canopy tree, subcanopy tree, shrub, ground layer); iii) the diameter of the smallest remaining woody twigs elevated 1–2 m above ground, based on a mean of 10 replicates.

Vegetation structure variables and, where possible, pre-fire structure were recorded at the time of survey in all subplots combined. Vegetation height in meters (lower, upper, and average estimate) and percentage projective cover were recorded for four vertical strata where present (canopy tree, subcanopy tree, shrub and ground layer). Pre-fire structure was inferred from scorched remains. Trait values of plant species were recorded in each subplot separately. In each plot, two traits that can be directly observed in single visits (regenerative organs, *surv4*, and seedbank type, *germ1*) were recorded for all species using the controlled vocabulary of the traits. Individuals of each species were divided into two groups: (1) present above ground during the fire; and (2) recruited as seedlings after fire. For the first group, we counted the number of individuals that survived unburnt (fully or partly; “adults_unburnt” in quadrat samples table in Online-only Figure 10), those that were fully burnt and resprouted from regenerative organs (“resprouts_live”) and reproducing or not (“resprouts_reproductive”), those killed outright by the fire (“resprouts_kill”), and those that burnt, resprouted and later died after fire (“resprouts_died”). For individuals recruited after fire, we counted living individuals (“recruits_live”) and whether reproductive or not (“recruits_reproductive”) and individuals that had died prior to survey (where observable as dead remains; “recruits_died”). For some species, the counts were discriminated by life stage (adults or juveniles) or the type of scorch (basal or partial vs. full canopy scorch). These observations form the basis for calculation of some species traits (examples *repr3* and *repr3a*, etc.) and for quantification of the local response of a species to different fire events.

We recorded data from each visit in a field work proforma (see Online-only Figure 11). We then transcribed data from all visits in each survey to spreadsheets for each table (location and visit descriptions, fire history, vegetation structure, and floristic plot data).

Customised scripts were written in Python to automate the importation of field data from the spreadsheets into the database. These scripts are available for download (see Code availability section), and are documented in a Jupyter Notebook file format with comments on each step of the process detailing the approaches used to accommodate slight differences in the input data files^37^.

### Data input - published records

We considered two major sources of published plant trait information that were readily available and would allow automatic import of records for each species by applying simple transcription rules to match the reported values into the controlled vocabularies developed in FEDB for each trait. We refer to these as *secondary sources*, because they compile information from other published or unpublished sources (which we call here *original* or *primary sources* for simplicity, although they do not always refer to a direct field observation).

The first source, the New South Wales Flora Fire Response Database (NSW FFRDB, version 2.1)^18^, is an unpublished product of the Department of Climate Change, Energy, the Environment and Water, New South Wales, Australia. The species-level traits from multiple sources were compiled in February 2010 and last validated in May 2014. The dataset and its documentation was previously available online, and currently available on request to NSW DCCEEW data broker (see NSW Government’s Open Data Policy http://data.nsw.gov.au/nsw-government-open-data-policy). This data source consists of a spreadsheet with two partially linked tables. The ‘SpeciesData’ table includes one row per species, with traits based on existing sources^11,16^ and summarised in multiple columns. Each cell of this table may contain one or more values, links to primary data sources stored in the second table, and additional information coded as formatting rules (colours and font features). Links to the data source table (‘References’ worksheet) are either hyperlinks to a cell number or textual references to a reference code. This composite source contains information on 26 species traits for 3,086 taxonomic units and includes a list of ca. 248 data sources (some duplicated). The data refer to plant taxa (species or other taxonomic units) that occur in New South Wales, Australia. From this source, we selected 12 traits that we could match to the traits defined in Table 1 in this descriptor.

The second source was AusTraits, a transformative database, containing standardised records of measurements on the traits of Australia’s plant species^23^. Version 6.0.0 of the AusTraits data is compiled from 364 datasets, includes 497 plant traits and 33,494 taxa for all Australia^38^. From this source, we selected five traits that we could match to the trait defined in Table 1, and species found in New South Wales (according to the BioNet Atlas).

The compiled AusTraits dataset has a similar structure for all traits. Each record refers to a trait observed for a single species and the basis of the record can be an individual, a “population” (from a particular sample) or a mean value for the species. Some records are associated with a sampling context, that describe sampling methods or temporal characteristics, and some records include spatial data describing sampling location.

Data entry of existing records was automated using customised scripts written in Python. Each script is documented in Jupyter Notebook file format with comments on each step of the process^37^. The scripts read and filter input data from NSW FFRDB or AusTraits and then transform the input value (raw value) into a normalised or ‘norm’ value (for categorical) or triplet of values (for numeric traits) using specific transcription rules for each input data source and trait in the Fire Ecology Database as defined above. For qualitative traits the raw values are mapped to a controlled vocabulary of accepted values following simple rules. For quantitative traits we used a triplet of values to describe uncertainty in the input raw values. If raw values include a single number it is taken as a best estimate, but if the raw value includes a range specified by minimum and maximum values, these are recorded as lower and upper bounds. The triplet of values (best, lower, upper) can be interpreted as a fuzzy number^34^.

Due to the nature of the data sources, the import scripts are tailored to the input data format and values. However, scripts can be adapted and customised to import other sources in the future. For NSW FFRD data the import script reads the data for each column of the ‘SpeciesData’ worksheet and transforms each cell into one or more records per species, and then inserts the records in the relevant tables of the database. The original sources were linked to each record whenever possible. For the compiled AusTraits dataset the import script was used to read individual records and add them into the Fire Ecology Database with a link to the original source. After importing records from both sources we flagged duplicated or redundant records (records sharing same trait information and same primary source, or crossreferencing between two secondary sources). These duplicates are ignored in exports, queries and analysis but still available in the database for data curation purposes.

## Data Records

Static versions of the Fire Ecology Database, including version 1.1 used in this descriptor, are available via FigShare or OSF in three different formats: (1) a complete export of the database structure and data as an SQL dump^39^; (2) analysis-ready data frames for use in R^40^; and (3) a summary export of traits and records per species in tabular formats using XLSX and CSV file formats^41^.

Data is released under a CC-BY license enabling reuse with attribution – being a citation of this descriptor and, where possible, original sources. The data with metadata and other descriptive information has been deposited within Figshare and OSF, consistent with FAIR principles^42^.

As an evolving data product, successive versions of FEDB are being released, containing updates and corrections. Versions are labeled using semantic versioning to indicate the change between versions. Versions prior to 1.1 have not been peer reviewed.

Contributions to the Fire Ecology Database are welcome and should be sent to the editor (D.A.K.).

### Data coverage

**Fig. 3:**
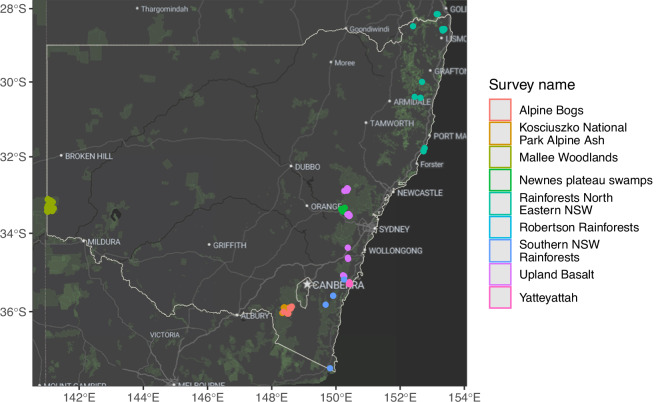
Geographic representation of records in the database. Location of sites where field observations were made for input to the fieldwork data stream (Online-only Figure 10). Basemap from Stadia Maps https://stadiamaps.com and Open Street Map https://www.openstreetmap.org.

**Fig. 4:**
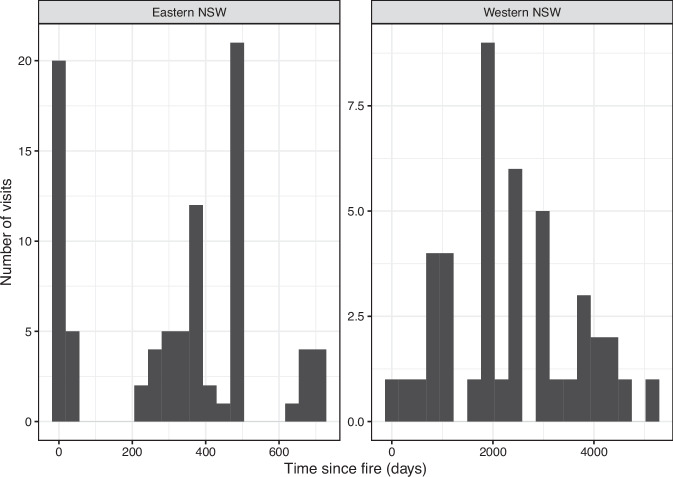
Timing of field visits in relation to time since fire for post-fire field surveys in eastern New South Wales, sampled between October 2019 and December 2021 after extensive bushfires during the 2019/2020 summer; and time series of field observations in western New South Wales conducted between 2007 and 2018 after experimental fire treatments.

Eight surveys conducted in humid coastal and tableland areas of eastern New South Wales between 2020 and 2022 sampled contrasting ecosystem types (three major types of rainforests, wet and dry eucalypt forests, heathlands, temperate and alpine peatlands) that were burnt in extensive bushfires across eastern Australia during the 2019/2020 summer (Fig. 3). Most of these sites were visited a few months after fire and some were visited multiple times, primarily in temperate heathlands (Fig. 4). The data from mallee woodlands in western New South Wales (Fig. 3) sampled vegetation at sites burnt in different years during multiple visits spanning a decade, a longer time frame than those sampled in eastern New South Wales (Fig. 4).

**Fig. 5:**
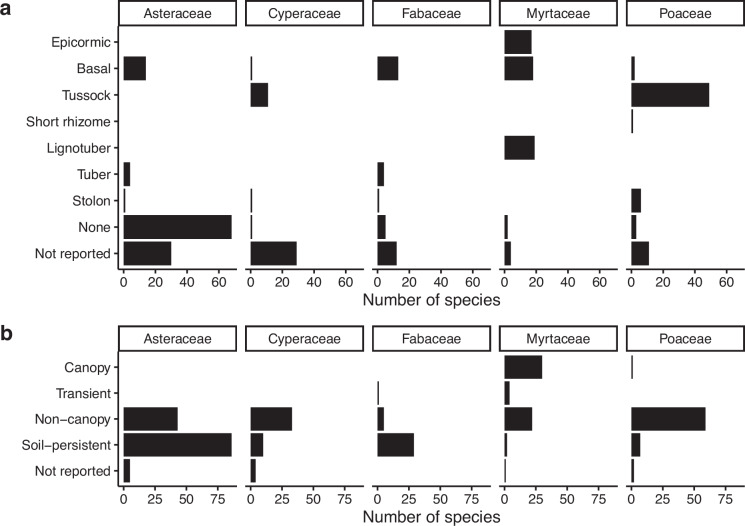
Resprout organs and seedbank type reported in the field observation data for the five most frequently recorded families. Bars represent number of distinct species for each category of: (**a**) the regenerative organ (surv4), and (**b**) the seedbank type (germ1). Species with multiple organs or seedbank types contribute to more than one category.

**Fig. 6:**
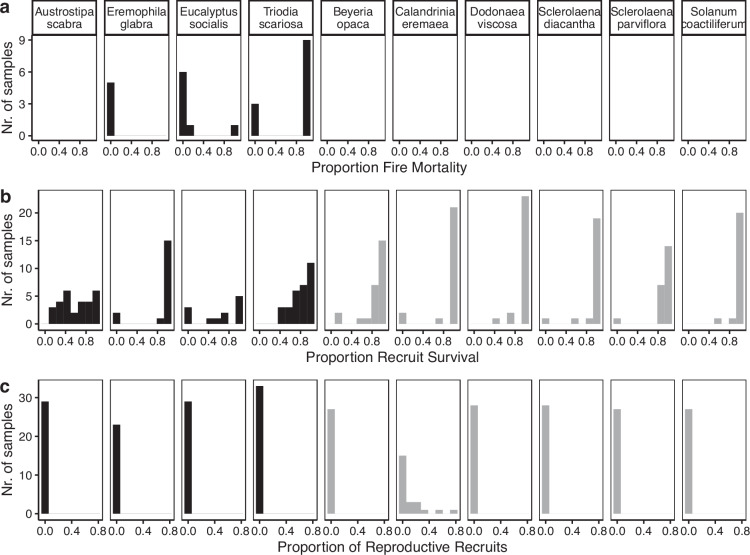
Distribution of the proportion of fire mortality (**a**), recruit survival (**b**), and reproductive recruits (**c**) calculated for the 10 species with the highest number of records in the mallee woodlands survey. The first four species on the left side are considered resprouters (black bars) and the others are considered obligate seeders (grey bars). Only sites sampled within the first three years since the last fire were included in the calculation of these metrics.

Data from the field surveys can be extracted and summarised to examine patterns in species traits and population responses. For example the five most speciose Families (Asteraceae, Poaceae, Myrtaceae, Cyperaceae and Fabaceae) in the field samples across all surveys show different resprouting and seedbank strategies (*surv4* and *germ1*, see Fig. 5). Within a species, the responses are variable between field sites visited within the first three years following a fire. Figure 6 shows three metrics calculated for the 10 most frequent species in the mallee woodlands survey. For resprouting species we calculated fire mortality as the proportion of fire killed individuals vs. the total number of individuals estimated to be present during the fire (including live, killed by fire or died post-fire). For both seeders and resprouters, the recruit survival is calculated as the proportion of live recruits to the total number of recruits estimated to have emerged after the fire (including live and dead recruits), and recruit reproduction as the proportion of reproductive recruits to the number of live recruits.

**Fig. 7:**
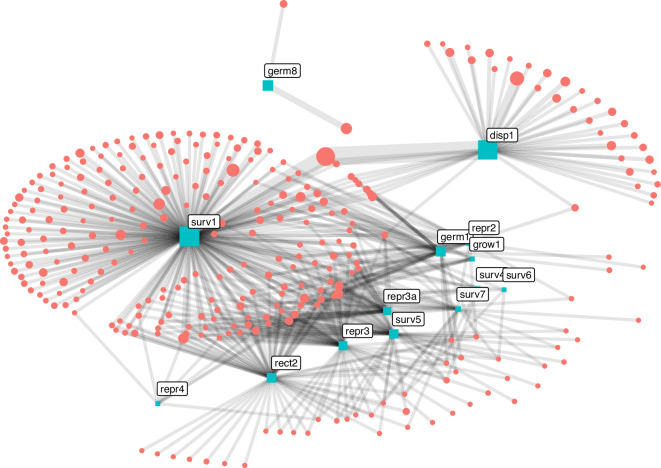
Graph of traits (labelled rectangles) per existing sources (circles) used to document 14 fire-related traits. Size of rectangles and circles is proportional to number of species with data from each trait/source.

**Fig. 8:**
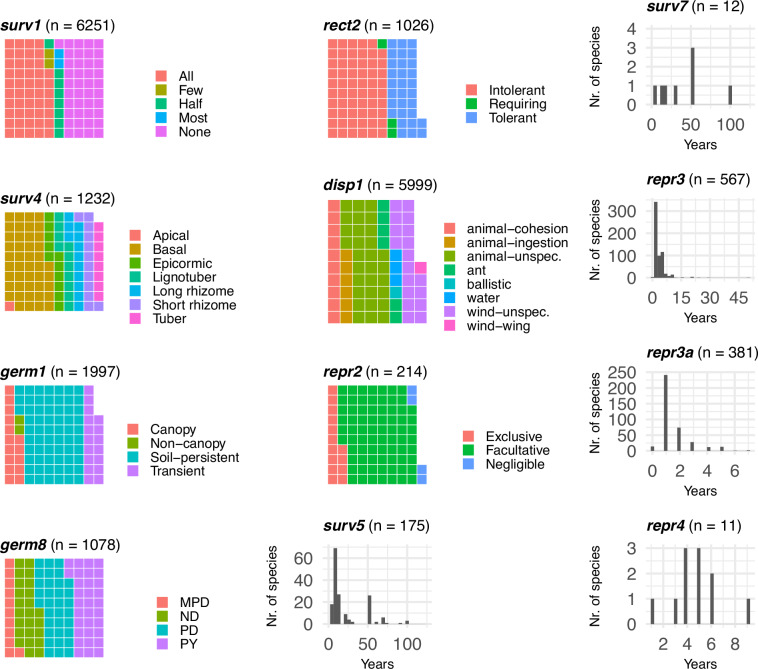
Visual summaries of trait values of 12 fire-related traits. Each summary is labelled with the trait code (see Table 1) and the number of species with valid records (n). For categorical variables we use waffle plots with different colours representing different categories and the number of squares proportional to the number of species in each category. Species with multiple entries might contribute to more than one category. For quantitative traits the histogram show the frequency of the values (based on the best estimate value for each record). Plots for traits surv6 and grow1 not shown due to small sample size (n < 10).

Our second data stream considers records of data or information that has already been aggregated and interpreted. This can originate from research literature, but can also come from expert elicitation or more informal sources. These data sources often refer to summaries for a species or entire populations, and apply different methods of observation and inference. For this initial release, we explored two existing compilations and used automated scripts in order to extract known records and transform the values reported in each source into common normalised values for qualitative and quantitative traits. We expect that additional data sources can be added directly to the database in future releases. The data imported through this stream includes 301 references and authors contributing information for our 14 priority traits. A graph representation of the links between references, traits and species (Fig. 7) shows that 167 specialised references provide information about single traits for one or multiple species, with the largest proportion contributing to one of the two better documented traits (‘surv1’ and ‘disp1’). Only 32 central references provide data for four or more traits and multiple species. Some germination and dispersal traits have a different suite of sources to other traits. A visual summary of trait values for categorical and numeric traits from this data stream is given in Fig. 8.

**Fig. 9:**
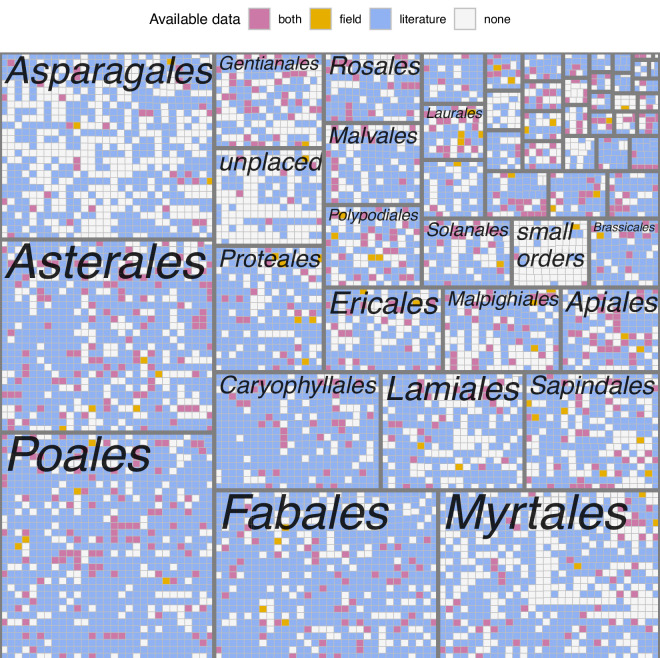
Taxonomic representation of records in the database. Larger rectangles represent plant orders, and each small rectangle represents a species within the order. Rectangles are filled according to the source of information available in the database for each species. Labels for 37 orders with < 200 species not shown.

Combining all these data sources, we have records for at least one fire-related trait for 5,573 native plant species in New South Wales, including primary field observations for 806 of them. Most of the larger plant orders and families are well represented in the database, with 77.7% of the genera having at least one species reported, but smaller orders show larger gaps in knowledge (Fig. 9).

## Technical Validation

We implemented several strategies along the data pipeline to maintain data quality, and document uncertainty in the database.

### Database governance

The database is governed on a voluntary basis, by an Editor (D.A.K.) and a Database Administrator (J.R.F.P.). Quality control of data and editorial procedures include:

- Contributor approval: Database users must request permission to become a database contributor.
- Editorial approval: Once a contributor sends data to the Editor, the data will be checked and if correctly formatted will be forward to the Database Administrator for data import and curation.
- User feedback: Data issues can be reported for any observation by email to the Editor or though the issues panel in the GitHub repository https://github.com/ces-unsw-edu-au/fireveg-db/issues.

Data import and curation protocols include the following features to maximise transparency, consistency, and the documentation and treatment of data uncertainty in the pipeline:

### Raw and normalised data

Raw input data and normalised data are retained in the system for each record of categorical variables. Records that cannot be normalised due to non-standard or conflicting information will have NULL value in the norm data field and are excluded from summaries. Errors during import are flagged in the comment field for manual review.

### Splitting conflated input records into multiple database entries

If a single input data entry includes data from multiple sources with ranges of values, this was translated to multiple data entries in the database, and a comment was added to help trace the origin of the record.

### Recording data lineage

We record the immediate source of the record (which may be a compilation of multiple sources, as in the NSW FFRD and AusTraits) and the original reference or source as reported.

### Weights

During data import each record is assigned a default weight. The weights can be modified during data curation, for example to down-weight dubious or redundant records, or to up-weight records that are considered more accurate. Reasons for changing weights can be documented in a unique comment field.

### Fuzzy number estimates for numeric trait values

For numerical traits we keep the raw input data and a triplet of normalised numeric values to represent a best estimate, a lower bound, and an upper bound, quantifying the range of variability and/or uncertainty in trait values.

### Controlled vocabularies

For categorical traits and methods of estimation we use predetermined and well described vocabularies of accepted values.

## Usage Notes

We created a webapp based on Python/Flask for data browsing and exploration. The webapp is available at http://fireecologyplants.net. This webapp connects to the SQL database and provides summaries by species, sites or traits and has been useful for interaction with data curators and potential data users. The code and documentation for the webapp is available in an OSF repository^43^. This includes instructions for setting up and running an instance in a cloud service provider. Future development of the web app will include features for data entry, batch import and data curation, when erroneous or missing data is flagged.

We also prepared a collection of Jupyter Notebooks describing common queries to the database and R code to create summaries and visualisations of the data. These are also available in the OSF project repository^44^.

## Figures and Tables

**Online-only Figure 10:**
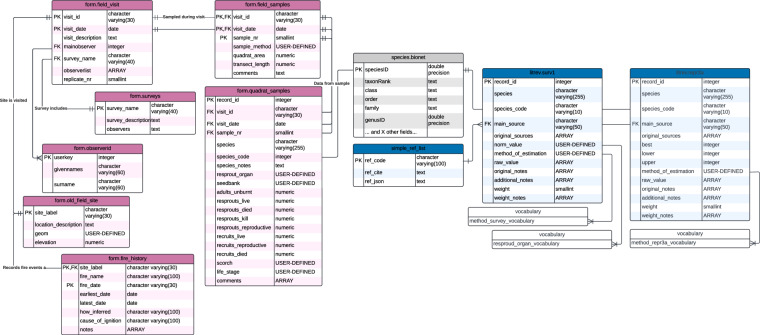
Entity Relationship Diagram for the database showing tables from its three schemata. The field form schema to the left includes seven tables. The existing sources schema to the right includes multiple tables for all traits, but only one categorical (surv1) and one numerical trait (repr3a) are shown.

**Online-only Figure 11:**
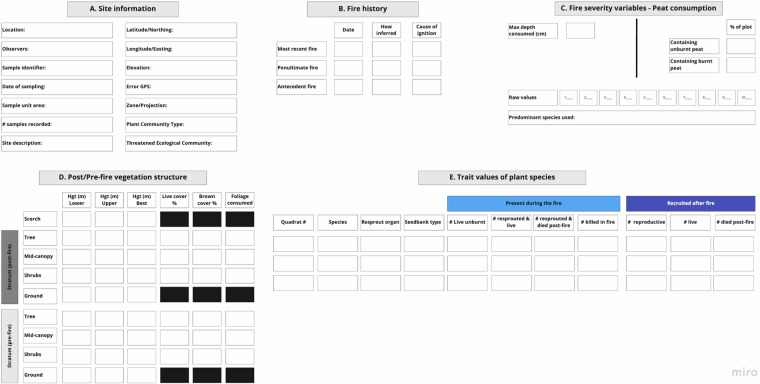
Example of field proforma.

## Data Availability

The database was implemented using postgresql and the sql code for creating tables, indices, types and constraints is available in the following repository: https://github.com/ces-unsw-edu-au/fireveg-db^45^. We used customised scripts for batch import of data from available data structures. The scripts were written in Python and documented using Jupyter Notebooks, they are available in the following repository: https://github.com/ces-unsw-edu-au/fireveg-db-imports^37^. The source code for the web app (using Python/Flask) is available at: https://github.com/ces-unsw-edu-au/fireveg-webapp^43^. Additional Python and R scripts for summarising and visualising the data, including the plots presented in here are available at https://github.com/ces-unsw-edu-au/fireveg-analysis^46^.

## Online-only Table

**Online-only Table 3:** Fire-related traits to describe responses of plant species to fire.

Trait name	Trait code	Description
*Dispersal*/*Seed*		
Propagule dispersal mode	**disp1**	Categorical. Propagule dispersal mode.
*Germination*/*Seed*		
Seedbank type	**germ1**	Categorical. Location & persistence of seedbank.
Heat-pulse-cued germination	germ2	Categorical. Germination response to heat pulse.
Smoke-cued germination	germ3	Categorical. Germination response to smoke treatments.
Heat shock-smoke interactive		
germination cue	germ4	Categorical. Germination response to combined heat and smoke treatments.
H50	germ5	Numerical. Minimum temperature of heat pulse (exposure time up to 10 mins) producing 50% germination from viable fraction.
H-max	germ6	Numerical. Temperature of heat pulse (exposure time up to 10 mins) producing maximum % germination from dormant fraction.
H-lethal	germ7	Numerical. Minimum temperature of heat pulse (exposure time up to 10 mins) at which all seeds are killed.
Seed dormancy type	**germ8**	Categorical. Seed dormancy type
TBA	germ9	Categorical. TBA
Postfire residual seedbank	germ10	Categorical. TBA
*Growth*/*Standing plant*		
Age to develop regenerative/resistance organs	**grow1**	Numerical. Developmental age for fire- regenerative or resistant organs (Age at which half an even-aged cohort can survive full scorch).
Age to reach maximum growth stage	grow2	Age of a cohort at which 50% of individuals reach their maximum growth stage
Maximum growth stage	grow2a	Taxon-specific definition of maximum growth stage (e.g. specify a minimum plant height, canopy diam. organ development (e.g. rhizome), dbh, presence of hollows, etc.)
Maximum bark thickness	grow3	Numeric. Maximum bark thickness [mm].
Maximum plant height	grow4	Numeric. Maximum plant height [m].
*Recruitment*/*Seedling*		
Postfire seedling recruitment	rect1	Categorical. The observed incidence of post-fire (within first 5 years after fire) seedling recruitment
Establishment pattern	**rect2**	Categorical. Seedling establishment pattern in relation to mature vegetation.
Seedling emergence phenology	rect3	Categorical. The month(s) of seedling emergence event. Enter either: i) square brackets with comma-separated first month, peak month and last month of seedling emergence event; or ii) single month of observed emergence if first, peak & last unknown)
Seedling emergence phenology — rainfall context	rect3a	Categorical. Occurrence of significant dry spell ( > 3 months with < 10 mm in any week) during the period between fire and seedling emergence
*Reproduction*/*Standing plant*		
Flowering time	repr1	Earliest (1st decile of individuals) and latest (10th decile of individuals) month flowering was observed
Post-fire flowering response	**repr2**	Categorical. Strength of flowering response after fire events.
Age at first flower production (from seed)	**repr3**	Numerical. Age at first flower production (years).
Time to first postfire reproduction (from resprouts)	**repr3a**	Numerical. Time to first reproduction after fire for resprouting taxa (years).
Maturation age	**repr4**	Numerical. Years taken for 50% of an even-aged cohort to produce their first viable seed.
Age at maximum seed production	repr5	Numeric. Age of a cohort at which seed production reaches a peak, before declining with age
Age of maximum seed bank size	repr6	Numeric. Age of a cohort at which the accumulated seedbank reaches a peak (usually derived from models of seedbank dynamics)
Resprout flowering response to summer fire	repr7a	Proportion of surviving resprouting individuals that produce a seed/spore crop within the first five years after a summer (Dec-Feb sth hemisphere) fire.
Flowering response to autumn fire	repr7b	Proportion of surviving resprouting individuals that produce a seed/spore crop within the first five years after an autumn (Mar-May sth hem-isphere) fire.
Flowering response to winter fire	repr7c	Proportion of surviving resprouting individuals that produce a seed/spore crop within the first five years after a winter (Jun-Aug sth hemi-sphere) fire.
Flowering response to spring fire	repr7d	Proportion of surviving resprouting individuals that produce a seed/spore crop within the first five years after a winter (Jun-Aug sth hemi-sphere) fire.
*Survival*/*Standing plant*		
Resprouting - full canopy scorch	**surv1**	Categorical. Proportion of individuals surviving & resprouting after full canopy scorch.
Basal scorch response (scorch height <50% of plant height)	surv2	Non-ordinal categories of survival response for plants subjected to basal scorch.
Survival - basal scorch	surv3	Ordinal categories of survival and resprouting proportions for plants subjected to LESS THAN 100% canopy scorch.
Regenerative organ	**surv4**	Categorical. Regenerative organ type.
Standing plant longevity (max)	**surv5**	Numerical. Maximum life span *in situ*.
*Survival*/*Seed*		
Seedbank half-life	**surv6**	Numerical. Time taken for half of an even-aged seed crop to die/decay *in situ*.
Seed longevity	**surv7**	Numerical. Maximum life span *in situ*.

Traits currently in the database in bold. TBA: trait definition and/or description under discussion, to be announced in later releases.
